# The barriers and facilitators that indigenous health workers experience in their workplace and communities in providing self-management support: a multiple case study

**DOI:** 10.1186/s12913-017-2265-5

**Published:** 2017-05-03

**Authors:** Jessica Conway, George Tsourtos, Sharon Lawn

**Affiliations:** 1 Flinders University, Adelaide, Australia; 2 Discipline of Public Health, Flinders University, Adelaide, Australia; 3 Flinders Human Behaviour and Health Research Unit, Department of Psychiatry, Flinders University, Margaret Tobin Centre, PO Box 2100, Adelaide, South Australia 5001 Australia

**Keywords:** Chronic condition management, Self-management support, Indigenous health worker, Flinders program, Closing the gap, implementation

## Abstract

**Background:**

The inequality in health outcomes between Indigenous (Throughout the paper, the term Indigenous will be used to represent both Aboriginal Australians and Torres Strait Islander Australians.) and non-Indigenous Australians continues to be a major public health issue. Chronic conditions are responsible for the majority of the gap in life expectancy for this population. Evidence suggests that chronic condition management models focusing on self-management have led to improved health outcomes in Indigenous populations. The Flinders Closing the Gap Program (FCTGP) is a chronic condition care planning tool which aims to engage Indigenous people in self-managing their chronic conditions. Indigenous health workers (IHWs) can provide culturally appropriate self-management support; however there is paucity in current literature describing specific barriers and facilitators that they may experience when attempting to deliver this support. This study aimed to explore IHWs’ perceptions of the effectiveness and appropriateness of the FCTGP, as an evidence-based example of self-management support, and to explore the barriers and facilitators that IHWs experience in their workplace and communities in providing self-management support.

**Methods:**

In-depth interviews were undertaken with five IHWs, drawn from five different states in Australia. Their selection was aided by key informants from the FCTGP training unit. Interviews were recorded and transcribed verbatim, and were analysed using thematic analysis.

**Results:**

The following themes were identified. IHWs reported that the FCTGP was appropriate, flexible and acceptable in their communities. Facilitators included factors improving client and worker empowerment, and activities around sharing knowledge. Barriers included competing priorities that clients experience relating to social determinants of health, and negative experiences within mainstream health services. IHW burnout from time pressures, lack of support, and high staff turnover were also considered important barriers.

**Conclusions:**

This study contributes an insight into the experiences of IHWs who are considered important stakeholders in implementation and sustainability of chronic condition management programs, including the FCTGP. Recommendations focus on supporting and supplementing the role of IHWs and identify the FCTGP as a facilitator in providing self-management support to a population with complex needs.

## Background

Chronic conditions are responsible for the majority of disability and death in Australia [1] causing a significant impact on quality of life of Australians and placing a strain on health and welfare services [2]. Chronic diseases affect Indigenous Australians at a disproportionately higher level than non-indigenous Australians [3]. Chronic disease occurs at earlier ages in Indigenous Australians, accounting for two thirds of the gap in mortality between Indigenous and non-Indigenous populations and contributing to early morbidity [4]. For example, in 2012–2013 Indigenous Australians were more likely to suffer asthma, hearing loss, heart and circulatory related chronic disease, and insulin resistance and diabetes mellitus than non-Indigenous Australians [3]. The greatest discrepancy was in diabetes and chronic kidney disease with Indigenous Australians three times more likely to suffer diabetes and more than seven times as likely to receive treatment for end-stage kidney failure [2]. This inequitable situation calls for more effective healthcare models that improve chronic condition management (CCM) for Indigenous populations. However, Indigenous populations can experience exclusion and marginalisation from mainstream, Western bio-medical models of healthcare. The reasons for this have been written about extensively and relate to cultural differences in health belief systems and the impacts of colonisation and consequent effects of collective trauma arising from this for Indigenous populations [5].

CCM models that promote collaboration, multi-disciplinary communication and patient engagement have headed an international movement in primary care medicine over the last decade [6]. Self-management support (SMS), provided by health workers to patients, has been a key aspect of these models [6, 7]). Self-management is ‘being actively involved in managing one’s own chronic condition, and not simply receiving expert opinion from a health educator’ ([8], p. 172). It involves ‘a broad set of attitudes, behaviours and skills’ which assist people to manage ‘the impact of the disease or condition on all aspects of living’ ([9] p. 7, 10, 11) Yet, there is concern that these concepts must also be sensitive to the worldviews of Indigenous peoples, particularly because they are underpinned by assumptions of empowerment and engagement [10]. Models of care must involve an awareness of the social, emotional and economic context of Indigenous groups with sensitivity to the consequences of grief and loss, experienced by them, associated with Australia’s history of European colonisation [11]. The context for this is one of a history of dispossession, racism and a denial of basic human rights following European arrival to Australia in order to seek new land and wealth [12]. The dispossession of land and rights continued throughout the nineteenth and twentieth centuries with policies disregarding the rights of traditional owners and notably, the failure of the Australian Constitution in 1901 to recognise Indigenous Australians as citizens [12]. The invasion of Europeans was also hallmarked with the relocation of Indigenous people to missions and reserves, and the traumatic separation of Indigenous children from their families, known as the ‘Stolen Generation’ [13]. This process prevented the ‘Stolen Generation’ from acquiring their native language and culture, and denying them a spiritual connection with land and people [13]. All of which have contributed to the ongoing impairment of the health and social wellbeing of Indigenous Australians today. The causal links between ongoing institutionalised racism, cultural insensitivity, continued socioeconomic disadvantage and health have only been acknowledged in the last two decades [12, 14]. The negative impact that European invasion has had on diminishing empowerment and self-determination in Indigenous Australians is pivotal in understanding the importance of re-establishing personal agency and control for Indigenous people in relation to improved health outcomes [10].

The Flinders Chronic Condition Management Program (Flinders Program) has been evolving since its foundation during the seminal South Australian HealthPlus coordinated care trials in 1997–99 [15], which were part of a national initiative for researching options on Australian healthcare reformation to better care for chronic conditions. The program provided a holistic CCM intervention tailored to the individual, with specific tools designed to achieve more patient-centred assessment of care needs, improved coordination of care between care providers, and stimulate engagement and collaboration with patients to promote better self-management [16]. These tools include the Partners in Health Scale (PIH), Cue and Response interview (C&R), Problem and Goal Assessment (P&G) and Chronic Condition Self-Management Care Plan, which are described in more detail in Table 1 below.

**Table 1:** Summary of the Flinders Program tools [17]

Flinders Program tools
1) The Partners in Health Scale (PIH): A patient Likert-rated validated questionnaire informed by the WHO and Australian National Chronic Disease Strategy principles of self-management [59, 60] It enables measurement of perceived change over time where 0 = less favourable and 8 = more favourable self-management capacity. Self-management rated capacities include: knowledge of condition and treatments; quality of relationships with healthcare providers; actions taken to monitor and respond to signs and symptoms; access to services and supports; physical, social and emotional impacts, and lifestyle factors.
2) The Cue and Response Interview (C&R): An adjunct to the PIH using open-ended questions or cues to explore the patient’s responses to the PIH in more depth, with the patient and worker comparing their Likert-ratings to identify agreed good self-management, agreed issues that need to be addressed, and any discrepancies in views that can then be discussed as part of formulation of a self-management care plan. It enables the strengths and barriers to self-management to be explored, and checks assumptions that either the worker or patient may have, as part of a motivational process.
3) The Problems and Goals (P&G) Assessment Defines a problem statement from the patient’s perspective (the problem, its impact and how it makes them feel) and identifies specific, measurable, achievable, realistic and timely (SMART) goals that they can work towards. It is Likert-rated, allowing measurement of progress over time where 0 = not a problem and 8 = a significant problem; and goal statements: 0 = no progress towards achievement and 8 = achieved.
4) Self-Management Care Plan: Includes self-management issues, aims, steps to achieve them, who is responsible and date for review.

The Flinders Program tools were adapted for use with Indigenous Australians within the Closing the Gap initiative, as part of a series of national consultations to ensure their cultural sensitivity. They became known as the Flinders Closing the Gap Program (FCTGP) Tools, and later, ‘My Health Story’. For this adaptation process, the PIH and C&R were combined into a single encounter, terming it ‘My Strengths, Needs and Worries’ , and the P&G termed ‘My Main Worry and My Goal’ [17]. Evidence from evaluation of care planning in Indigenous communities with IHWs has been promising [18, 19]. Many features of this version of the Flinders Program tools have been applied to the generic Flinders Program. Current literature suggests the generic Flinders Program is acceptable and appropriate for Indigenous people; that it facilitates communication between patients and providers, empowers patients and helps identify health problems. Current evidence reflects that IHWs are integral as providers of holistic and culturally safe care to Indigenous Australians, providing a milieu for better access to health care for Indigenous people due to shared cultural understandings [20, 21]. For this reason, building the capacity of the Indigenous health workforce is described as a key strategy within current state and federal Indigenous health policies [18, 22–24] and IHW participation in decision-making in and around health service provision is considered crucial to implementation of primary health care interventions such as CCM [25–29]. Yet, there is a lack of understanding of the perspectives of providers such as IHWs in using these tools to support Indigenous Australians in CCM [8, 18, 30, 31]. The aim of this study is therefore to describe the barriers and facilitators that IHWs experience in providing SMS to Indigenous Australians, as well as their perception of the appropriateness and effectiveness of the FCTGP in aiding SMS, in order to inform and support CCM strategies in this population from these crucial stakeholders.

## Methods

Case study methodology underlies the way we answered the research question: ‘What are the facilitators and barriers that IHWs experience in providing SMS?’. Within the realms of this current study, the cases in question are the IHWs and their experience of providing SMS to Indigenous Australians using the FCTGP. Case studies have been described by Stake ([32], p 238) as ‘the intrinsic study of a valued particular’ , where the object of study is within ‘a specific, unique, bounded system’. Within the realms of this current study, the case in question is the IHW’s experience in providing SMS to Indigenous Australians using the FCTGP Tools in the complex cultural, political and social contexts that they live and work within.

Also, in evaluating a program such as chronic condition management, case studies may help to describe an intervention in the real world context and explain the causal links which would otherwise be too complex for survey methods [32] (see also Yin [33]). This is particularly important when exploring facilitators or barriers to a program, where the how and why of whether or not a particular program is efficacious and appropriate. This is what was explored in dialogues with IHWs [33]. Multiple cases were selected because they assisted in advancing an understanding in relation to the research question [33], because each case illustrated different aspects of the issue in which the researcher is interested in order to gain a better understanding of the issue (the barriers and facilitators to providing self-management). Ideally the cases are observed from multiple forms of data. The cases largely relied on data from the interviews with IHWs and notes taken throughout the interviews from observations made during the interview on body language and tone. This aimed to supplement the data.

The target population involved health workers who identified as being Indigenous Australians and were providing SMS to Indigenous Australian clients living with chronic conditions. An initial goal of 10–12 cases were sought as this was thought likely to provide sufficient data with enough literal and theoretical replications for data analysis. Initial recruitment was attempted in the form of an email sent to 201 IHWs who were registered on a database through the FCTGP. Of these there was only one response (Sandy).

Following a discussion with the research team, a different approach was sought with purposive sampling involving an invitation by email and telephone from people who had been recommended by Key Informants to provide different perspectives. Key informants included the Education and Training Officer for the FCTGP at Flinders University, and the National Business Implementation Manager for the FCTGP. Suggested cases were of IHWs who had been observed to either succeed or face difficulties in implementing SMS. Key Informant 1 also recommended managers of Aboriginal Medical Services who also were able to suggest suitable IHWs (likely to be interested in participating, and had either experienced difficulties or successes in chronic condition management). All participants responded to this invitation via email and gave consent before being interviewed. The number of cases recruited was partly determined by time restrictions imposed on the interviewer. The chosen five cases, however, provided literal and theoretical replications of data for analysis with a mix of male (two) and female (three) participants from rural and urban centres in five different states [33].

In-depth interviews were used to investigate the perspectives of IHWs on using the FCTGP to provide SMS to Indigenous clients of their health services. Using in-depth interviewing is regarded as a culturally appropriate means to gather information in a population familiar with using story-telling, or ‘yarning’ as a means to exchange information [21, 29, 34, 35]. The formation of interview questions was based on literature review findings and the study aims, and further developed and revised during data collection. Given the lack of studies specifically asking IHWs about the barriers and facilitators to providing SMS, questions initially took the form of working hypotheses, which underwent constant review following each interview. The interview questions used to guide the interviews is provided in a box below (Table 2). Interviews occurred either face-to-face (*n* = 1) or via telephone due to distance from the researcher (located in other states of Australia) (*n* = 4). Inaccuracies due to poor recall of the interview were minimised by the lead researcher audio-recording the interviews and transcribing them verbatim. Each participant was interviewed once for approximately 1 h. All interviews occurred in late 2015 and were conducted in English. The interviewer was a public health clinician with a medical degree and interest in Indigenous health issues who undertook this research as part of a Master of Public Health degree. They had no prior relationship with the participants.

**Table 2:** Interview question guide

Interview questions
Flinders Program – Preliminary Questions Tell me a bit about your role at the moment, what do you do from a day to day basis? When did you first hear about the Flinders’ Program? How did you come to do the training? How did you find the training?
Logistics and application of the tools How do you find using the tools such as My Health Story at work? How do you find explaining the tools to clients/patients? How many parts of the care planning process were used? Do you think the clients like the tools? Do you feel the tools are appropriate to your patients needs? How much was the client able to participate and form their own goals? Did they need prompting? Did it empower/build their capacity to self-manage? (if so, how so, and if not, what are your thoughts about why?)
Self-management as a concept/personal attitudes/values towards self-management What do you think are the good/positive parts of self-management/the tools? Do you think there are any negatives to self-management/the tools? What do you think of the My Health Story booklet? Has exposure to these tools/self-management changed the way you work with people? How? Why?
System supports Were you able to access follow-up support after the workshop? How confident do you feel using the tools? Is there any ongoing support, training or supervision of its use?
Self-management in the workplace How has your workplace implemented the Closing the Gap strategy? What have been the issues? What has worked well? Not so well? Why? Do you feel supported by your workplace in supplying self-management support? Do you feel that using the tools helps to improve continuity of care? Do you feel you have adequate time to use the tools? How has the IT system at your workplace helped you use the tools?

Member checking occurred whereby the data interpretation was shared with the participants to ensure accurate representation of the data collected [36]. This also included the opportunity to add further perspectives and clarification, which improved validity. Four out of five participants provided feedback and agreement with the transcript within a week of receiving transcripts. All felt their ideas were accurately represented in text.

Data analysis for this study was performed using thematic analysis. During analysis, reading and familiarisation of the data occurred during transcription. All three researchers coded the first two interviews separately to improve validity of commonly identified codes, and helped prevent codes being missed. The three researchers resolved any differences in how transcripts were coded by meeting to discuss the codes and explain their reasoning and thinking processes they used for interpretation of the meaning of the data. This shared discussion served to establish a clear set of guiding ideas for how further interviews would be coded. It also enhanced the depth of interpretation and enabled the researchers to reach overall agreement. Complete coding was performed manually using mind mapping software, Mind Maple Inc. [37], and spreadsheets to group themes and show their relationships. Final themes were decided on considering the themes’ and subthemes’ hierarchical and lateral relationships. The final stages of analysis involved writing about themes and subthemes and using direct references from the data to provide evidence and maintain the voice of the participants. Notes taken on emphasised words, pauses and changes in vocal tone during interviews provided more opportunity for data triangulation [38].

## Results

**Fig. 1:**
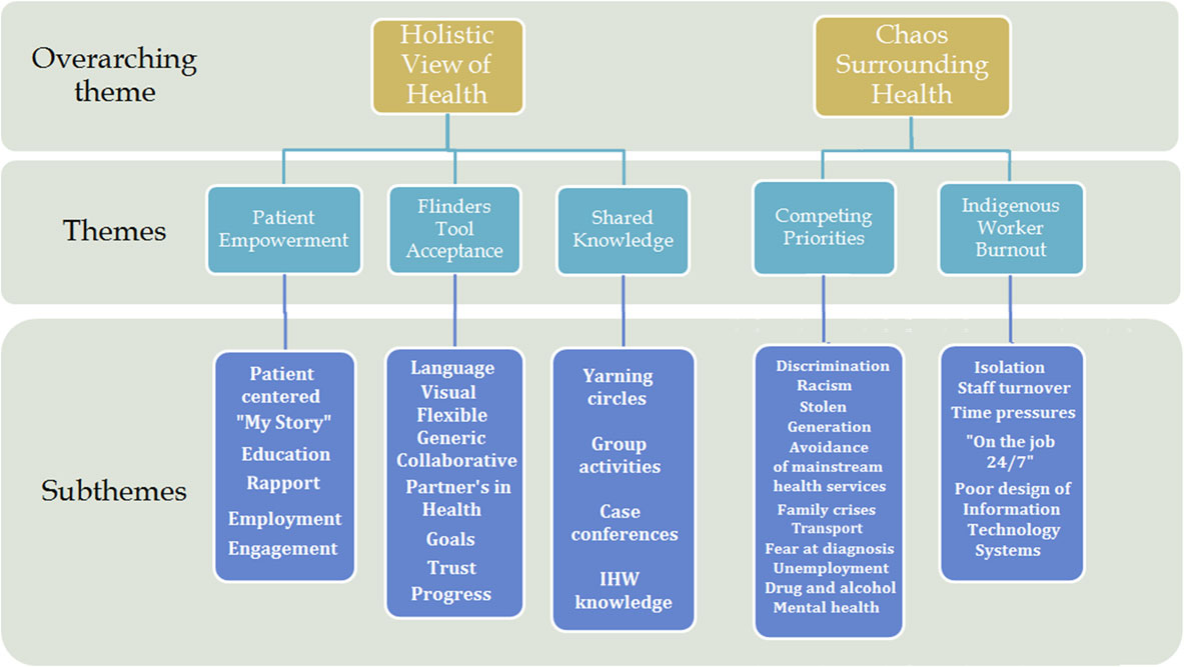
Themes

**Fig. 2:**
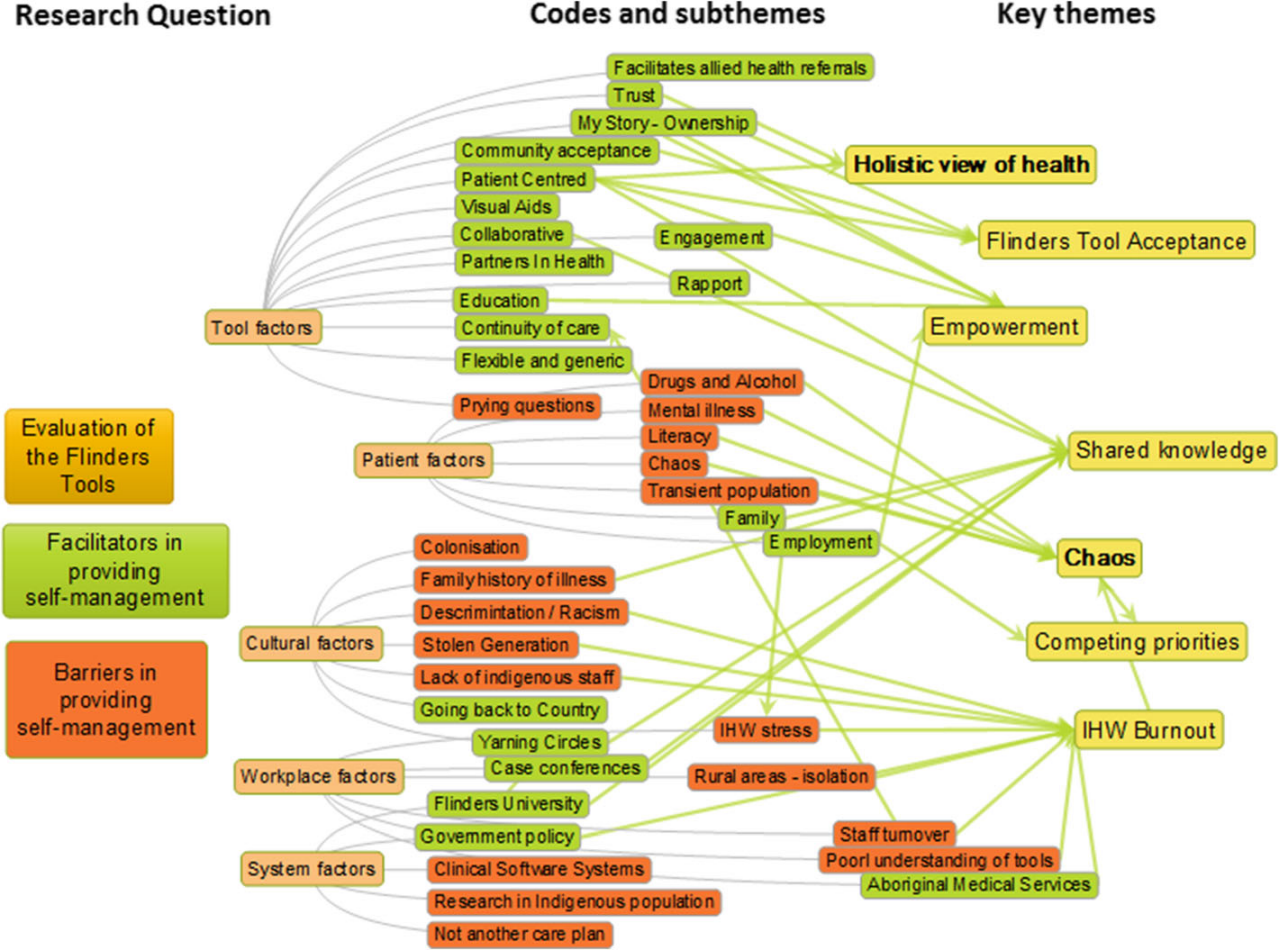
Interrelation of themes

Two overarching themes were identified, including ‘Holistic View of Health’ , and ‘Chaos Surrounding Health’. Within the holistic view of health, main themes included ‘Patient Empowerment’ , ‘Acceptance of Flinders Tools’ and ‘Shared Knowledge’. These themes are intertwined with common features; in particular, how the Flinders Tools assist patient empowerment, and sharing of knowledge. Within the chaos surrounding health, the two main themes included ‘Competing Priorities’ which feature the social determinants of health, and ‘IHW Burnout’. A diagram of the themes and the relationships between them is provided (see Figs. 1 and 2). Fig. 2 represents a mind map showing the complexity of inter-related themes from which final themes were chosen and provided more clearly in Fig. 1. It should be noted that whilst some subthemes may appear as facilitators, such as family providing support and cultural security, they may also appear as a barrier in the context of familial disease causing fear of diagnosis. This underlines the importance of recognising a dynamic context in understanding how certain subthemes may be on one hand a facilitator and another, a barrier. An effort has been made to clarify between these in the discussion. Participants have been given pseudonyms to maintain confidentiality. General workplace-related information about participants is also provided in Table 3 below.

**Table 3:** Participant vocational background

Participants
Greg is a Medicare Local IHW from rural New South Wales (NSW) working via an Aboriginal Medical Service (AMS). He has worked in welfare for 30 years, and has been a care co-ordinator for 3 years.
Jane works as a care-co-ordinator for Closing the Gap in Tasmania, funded by Medicare Local. She is a registered nurse, and has been care co-ordinating for 5 years, and has been a trainer in the FCTGP tools for 3 years.
Trevor is an IHW working through a GP Plus branch AMS in urban SA and has done so for the last 7 years. He was introduced to FCTGP 6 months ago.
Sandy is an IHW working in rural Victoria who has been doing care co-ordination for the past 3 years, and worked as Indigenous project officer in her health service for 8 years.
Brenda is an IHW working for the past 7 years as a care coordinator for the Aboriginal and Torres Strait Islander Chronic Disease Program at her AMS in rural Queensland. She has worked in community health for 10 years.

### Theme 1: patient empowerment

One of the key findings is that client empowerment was a facilitator in the success of self-management. Empowerment, in the participants’ terms, meant being able to tell their own story, identify their own problems and goals, and be given opportunities to take control of their conditions through education and support. There was more open disclosure of health issues compared with previous care plans, as Sandy notes ‘*It’s their life, their life story, and they’re telling it how they want to tell it … with other care plans, it’s very clinical so, they’re not delivering the story.’* Jane also reports that the FCTGP tools built clients’ capacity to self-manage through empowerment in a greater capacity than traditional care plans where patients have a sense of ownership over the design of their goals and meeting their needs:

*They’re designing the trajectory…[It] empowers them to think … it is in my words, it is my story, … they are my goals, so then it is reflective of what their actual needs are, … it definitely has the potential to be more successful in them being in more control than some of the more conventional type care plans.*

Participants describe a movement away from a ‘medicalised model’ of care, which increased patient motivation. As Jane describes, *‘We’re moving people more slowly and successfully towards their goals, coz they’re sort of seeing … they run their life and their conditions rather than the other way around’ … ‘Once you actually start to see some results and evidence that you’re making an impact… they’re far more likely to continue on that path’.* Multiple participants noted that the tools were able to assist patients to identify gaps in their knowledge about their condition’s signs and symptoms and use of medication.

Wendy felt strongly about issues surrounding welfare dependency and felt the self-management tools helped to combat that and lead to empowerment, rather than dependency:

*It’s making them more responsible for their own health, ….. We were finding up here that a lot of services were offering a saucepan if they got a health check done … that’s turning back the clocks and making them very welfare dependent, where this book is theirs, they can see where their health issues are, they can see what’s causing some of these problems, and they then work on it themselves.*

Here, Wendy described old models of bribery and reward as the antithesis to progression of health promotion and chronic condition management for Indigenous people, and welcomed the use of the My Health Story booklet.

Empowerment is linked to trust and further promotes engagement with the care planning process. Trust was integral to the process, and community education assisted in building trust, as Brenda explains ‘it’s just building that rapport, that trust with community members, to access and I guess that’s a lot of that education, community education… yarning circles’.

Trevor noted however that, in some instances, the tools were felt to be too detailed and prying, deterring patients: ‘*some thought it was a bit long, you know, you want to know everything about me, it’s just.. a lot of questions’*. This may reflect how a degree of rapport may be required before getting into the detailed elements of care planning.

Trevor also noted the importance of social determinants of health in providing empowerment for Indigenous Australians. In particular he identified the need for employment to give a sense of empowerment and control to improve health. ‘*Employment helps empower people you know, you give someone a helping hand and a start, a kick start, he will then get stronger and you hope for him to get stronger’.*

Trevor also described the difficulties faced by Indigenous people in gaining empowerment through employment in the face of racism and discrimination: ‘*The odds are put against us, just coz of the colour of our skin and the stereotype that we get…you know when something goes missing we gonna [going to] blame, why not blame the Aboriginal guy when it could be the white guy, you know.. but more likely it’s the black guy* …. *you get someone a job, you know, they’re self empowered. But to get that job …just really too, bloody hard’*.

### Theme 2: Flinders Program tools acceptance

The Flinders Program tools were described as appropriate in terms of their language and visuals. Greg and Brenda noted how patients could discuss issues in their own terms. The booklet was described as having clear language that minimised medical jargon, generic, flexible and highly adaptable to any condition and age group, as well as patients in transit who could take the book with them to the next clinic they visit*, as Sandy notes: That’s the other great thing about it… Someone might be just in transit, but we can go through the tools with them and then they can take the book with them …. and they can keep using it.*

All participants identified trust as pivotal in creating a collaborative partnership. The continuity of care provided by care planning helped to build trust, which in-turn lead to higher client retention and disclosure. Sandy notes: *It’s unearthing a lot of that underlying stuff that you don’t normally find out until you’ve been with a client for quite a long time. With this they start to open up, … and you’re starting to get to the core of the problem a lot quicker.*

### Theme 3: shared knowledge

Knowledge sharing was an important method for CCM. Ways of sharing knowledge included ‘yarning circles’, described by Brenda as an informal group meeting to share experiences and knowledge, which also builds trust and provides access to health information. Other group activities associated with improved motivation and success in self-management included walking or art groups, which fostered a sense of community.

Jane*: I find that if they can and they’re willing and able to access those groups even when it’s one on one like with strength to strength one on one, there’s still that sense of community, you know there are other people involved in the same program and they can share their stories, they tend to get more clarity around what their goal is and how they’re travelling with it and stay motivated.*

Greg also noted that walking groups had assisted one of his patients to quit smoking:

*‘we was going out bush and that and she was, well we started off and she was running out of breath when she was walking along and she’s had to have a rest. Then she said ‘oh I think this is from smoking’. And all that, and it’s ‘I need to cut down on me cigarettes’ and so she sort of said on her own and I said ‘that’s a good idea…cut down on your cigarettes’ And so they feel better for it, like, after they do activity they feel better for it.’*

Other modes of sharing knowledge were those within the staff members of the health clinics in the form of case conferences between a multi-disciplinary team. Family was an important support structure and motivator. Greg describes how being able to see their grandchildren grow up was a powerful motivator for many: *‘they seem to, you know, wanna [want to] get their life back … they wanna live to see their grandkids grow up’.* Family also share knowledge as evidenced by successful referrals from within families, as Jane notes ‘*I have found that most of our successful referrals have come from community, so whether it be word of mouth, someone’s has already been care co-ordinated and they will see that with family or friends’.*

### Theme 4: competing priorities

Competing priorities encompasses a broad theme of external barriers that IHW faced which impacted their ability to perform in their roles. There were also the competing priorities of their patients which detracted from their ability to engage with their health care goals as a priority. Barriers such as discrimination, lack of support structures, family crises, lack of transport and unemployment impacted on IHWs’ ability to care for their patients. Patients with drug and alcohol issues and or mental illness were described as a challenge to work with by some IHWs and this was embedded and exacerbated in a context of racism and disenfranchisement of Indigenous people, causing grief and trauma.

Greg reported that clients with mental health issues, largely relating to alcohol and drugs were challenging to work with: *‘Oh yeah you do get some clients, they don’t listen. Uh, like they say they’re gonna do this but they, it’s very hard to uh change their ways… clients who smoke and [drink] alcohol’*

When Greg was asked about which patients he felt were most difficult to provide self-management support with he described working with people from the ‘Stolen Generation’. The following quote is one particular example with whom he had difficulty working:

‘*because of their life… Like there is one lady… she is Stolen Generation…she was taken from her family when she was young. The department of community services come and took ‘em [them] and, they weren’t being cared for by their parents. And the father was an alcoholic so they removed the children and she was put in an institution, and she ended up with mental illness she had post traumatic stress disorder, and alcohol abuse and neglect’*

Alarmingly, it was identified that many Indigenous people avoid mainstream health services altogether due to discrimination, ultimately leading to poor access to health services. Poor access was also promulgated by fear of diagnosis of familial conditions in families, and a fear of the implications of diagnosis and failure to self-manage.

*Jane: I find, you get additional sense that obviously some of them have a degree of fear… of becoming really involved, because, particular for instance if you have a really low education level um you know, there’s that sense that they’re not gonna really have the ability to understand and grasp the technical side of things, so rather than attempt to sort of engage in that (self-management) they just allow it to be done to them, you know it’s in a more sort of a historical model of um, you know just taking on board what GPs have said you know just doing what you’re told … it can be a real eye opener for some people to realise that they can actually be really involved in making decisions around their care without having to absolutely know it inside out*

Client features associated with poor self-management related to either experiencing a traumatic past, including Stolen Generation, child abuse or neglect leading to mental illnesses such as depression and post-traumatic stress disorder. Brenda reported that one client didn’t open any Government letters, leading to missed specialist appointments, *‘especially around our elders who have a history of Stolen Generation and some can have that mistrust and miscommunication with non-Indigenous service providers’.*

One example of where care planning is used successfully in patients with mental health conditions is with Sandy, who started using FCTGP tools in place of general mental health care plans. She describes the mental health care plans as *sterile*, and the FCTG plans as easier and more effective: *‘it’s an easier program to use, it’s more straight forward, the community can understand it, there’s no stress for them … we’re actually finding a lot more stuff out that’s going on within the family by doing these care plans than what you do [with] the mental health care plans, because the mental health care plans are very, um, sterile, really.’*

Other competing tasks which detracted from patient care included system issues such as software design around data entry and referral processes depending on specific Medical Services. For example, on discussing documentation with Jane: *‘there’s a bit of double dipping and frustration from the worker’s point of view as well because they might be entering things on three or four different software systems … a lot of time banging words onto a computer when you could actually be out speaking to people’.*

On discussing barriers with Brenda she noted the inherent bombardment with referrals for patients newly diagnosed with diabetes: ‘*they do struggle with that self-management. It just gets too hard for them, you’ve got your diabetes educator, your dietician, your podiatrist, and uh, it just gets too hard for them and I find at times that they are overwhelmed with um all their appointments’*.

### Theme 5: indigenous health worker burnout

IHW stress was seen as a barrier to providing self-management and contributed to burn out and attrition, including feelings of isolation, which was more prevalent in rural and remote environments. There was a thirst for mentorship and camaraderie among IHWs who often worked under extreme time pressures, poorly designed information systems, high staff turnover, and with non-Indigenous workers who required cultural education to prevent cultural mishaps. Brenda describes ‘if there are any cultural issues that they need to be aware of, then it’s my job and (another health worker’s) job to advise’. All health workers felt time pressures were great: As Trevor commented on the process ‘*it was just way drawn out and we don’t have that much time with clients’ ,* Sandy reported ‘*I have to say that any problems that I encounter, there’s probably not enough time in the day for me to do what I need to do’* and Brenda described how they trained a hospital Indigenous Liaison Officer ‘*he said that within his role he is so busy, he doesn’t have time. We thought that would be ideal in his position, you know, you have the opportunity to sit down, but as they said…, they just don’t have the time’.*

The line between life and work was marred, with workers finding themselves on the job round-the-clock, for example they were expected to provide shelter for patients. Clear support structures to aid this are not routinely in place, and this was highlighted by Brenda:

*Brenda: In three years in a previous position I was burned out … I guess that it’s very important for Aboriginal and Torres Strait Islander staff members to have some support networks in place … I’ve found in my previous position which was more community model, you’re seen as one role in the community, 24/7 (laughs).. there’s no strategy to address that.*

*Brenda: The majority of workers live outside their community because of burnout… as I said you’re in one position in a community 24/7. [One worker] lived on the outskirts of her community, she was saying that in one night she could have seven people on mattresses.*

Staff turnover was identified as a barrier due to loss of continuity of care and trust for patients, which provided an expectation of failure to retain staff by community members.

*Jane: [Staff turnover has] a huge impact on a community and it’s very discouraging for them, change is what they expect and what disappoints them the most and as far as it’s very common for there to be a high turnover in some of these organisations of staff and there’s sort of that preconceived idea that you know “ohh we’ve got a newbie [person who is new to the organisation] on the block; she’ll be here for five minutes and she’ll be gone” and sadly we keep proving that theory correct.*

Jane also described the difficulties for new care co-ordinators to continue from where the previous worker left off as adding another layer to the complexity of staff turnover.

*It’s very difficult to maintain staff for any number of reasons. It’s a fairly challenging role, but it becomes incredibly frustrating for clients because they have to tell that story again and we give out inconsistent messages, which is not ideal.*

Also the lack of continuity affected the reviewing process and possibly inhibited the patient progression toward achieving their goals.

*Jane: It’s one thing to initiate the care plan but whether it is then followed up and reviewed … because we’ve had such a high turnover of care co-ordinators. And some that job share. And people move, and clients move around the state, so there are a number of factors that affect sort of the reviewing process but that’s probably not done as well as I think it could be.*

## Discussion

### Holistic view of health

The FCTGP Tools aim to create a culture of patient empowerment and responsibility by facilitating education and equipping patients with the means to make positive changes in their lives. The ‘whole of life’ view of health held by Indigenous communities is well documented [8, 39], and the holistic nature of the tools complement this [7]. There is an established need to value Indigenous knowledge and cultural beliefs in order to build trust and empowerment to promote engagement with awareness of the social context of racial discrimination, loss and grief associated with colonisation [18]. Appropriate language, a de-medicalised approach, and honest disclosure have been identified also as facilitating trust and communication in Indigenous people [35]. This is important as miscommunication and poor health education leads to a cycle of suspicion, blame and distrust of medications [29].

Sharing knowledge between patients, IHWs, other allied health staff, and families is considered a culturally appropriate approach to building community knowledge, and evidence from the literature supports this as a facilitator in CCM [29, 40]. The description of the use of yarning circles was limited in the literature [41–43], and may be a key recommendation for aiding SMS for IHWs. Local community group activities are described to promoting healthy activities and empowering patients [44, 45]. An area for future study could be on the impact on self-management of particular group activities. Chronic condition outcomes are improved when there is effective collaboration between health care professionals and patients [10, 46]. Information sharing should be timely and current in order to facilitate self-management support [47].

Family involvement is considered as a vital ingredient in care planning and self-management, and this must be acknowledged if self-management is to be successful [28]. Certainly, the importance of family in the structure of Indigenous communities is well documented [48–50], as is the role of family in providing support to patients with chronic conditions [50, 51]. However, it should also be acknowledged that family can also act as a barrier to self-management depending on the context [47, 52]. In the case of the IHW, family members can be a source of stress with obligations around caring for family and financial obligations [53].

IHW knowledge on Indigenous culture provided strength to their workplace, both in providing cultural knowledge to non-IHW and in supporting their patients. Poor cultural safety in mainstream health services limits and discourages access to health information and confidence in self-management [21, 28] and IHWs are working to combat discrimination in their daily work.

### Chaos surrounding health

This investigation found that access to Aboriginal Medical Services and IHWs is important for Indigenous Australians as poor cultural safety in mainstream health services limits and discourages patients’ access to disease information [28] and contributes to poor confidence in self-management [21]. Systematic problems relating to racism also have a negative impact of health, and this occurs directly in the health sector [51]. Effective culturally appropriate communication in the context of different world-views and concepts of physiology and disease can be challenging. However, the FCTGP tools acted as a facilitator in these settings and were described as culturally appropriate generic tools. Participants identified competing priorities relating to housing, employment and education as causing a barrier to assisting patients to self-manage; by perpetuating feelings of disempowerment and fear [54]. Poorer socioeconomic circumstances have been well described in the literature as a barrier to self-management [51–53, 55]. Social issues of poverty and poorer education opportunities are linked poor health literacy, which further marginalises Indigenous subgroups, which is more profound in non-Indigenous health services [51, 54, 56].

Other barriers to self-management identified by IHWs included factors affecting self-efficacy such as mental health issues, drugs and alcohol. However there have been cases where case-management has successfully been utilised to manage alcohol abuse [57], and another where motivational care planning and problem-solving activities were possible and promising for Indigenous patients with mental health issues [48]. One participant used the FCTGP tools specifically for patients with mental health problems in place of current mental health care plans, with great success. This was largely due to the holistic nature of the tools and de-medicalised language in comparison to conventional mental health care plans. This highlights how care planning is possible for patients with substance misuse or mental health problems.

IHW stress was a key reversible barrier found within this study. Evidence already suggests that often IHWs have limited support and are excluded from decision-making by other staff within their healthcare settings [27, 28, 53], leading to IHW burnout and attrition. Staff turnover impacts continuity of care, which can cause inconsistencies in care and patient demoralisation, and pressure on organisations to find new employees and train them. IHW support structures are a key requirement from this investigation. A lack of implementation champions for CCM programs to support IHWs in CCM and dedicated chronic disease positions compounded IHW stress. Adequate Indigenous representation in management and allied health positions was also identified as a barrier, and may improve implementation of CCM programs. Adequate staffing should also improve time pressures placed on IHWs and dedicated chronic condition positions, also recommended in implementation reviews [28, 30] should lead to a more sustainable approach within communities.

### Limitations and future recommendations

Recruitment difficulty meant only five participants were interviewed; more participants may have led to more theoretical replications. At the time of recruitment, many Indigenous health services were going through a period of flux, with federal uncertainty about funding to Indigenous health programs. Within such a climate, some IHWs might have been reluctant to fully engage with research. It is acknowledged that the majority of participants were supportive of the tools (*n* = 4), with only one who did not support them. It may have been of value to seek more participants who have had difficulties with the FCTGP tools, however all participants were able to identify barriers in their work and give feedback on where the tools may be improved. Future studies could work on improving processes for their recruitment, to bring different perspectives to the research. The methodology may also have been improved by the collection of data on each case from other sources. Triangulation of data could have been sought via the views of other members of the same clinic in the care co-ordination team and possibly the patients’ views. It is also acknowledged that it would have been useful to present to final paper to participants for their comments to further improve validity. Whilst this was offered, only one participant (Sandy) gave feedback on their transcript. The results of this study are limited to the Australian context, although several of the issues discussed have been shown as relevant to the experience of indigenous populations elsewhere and the IHWs who provide support to them. Recent reviews have suggested that chronic condition programs for Indigenous people from other countries share common barriers and enablers to implementation, and that a principal role for IHWs is one such enabler [58] Other studies including Canadian and New Zealand Indigenous chronic disease programs reveal that they have common features including the inclusion of community governance and a principal role for IHWs.

## Conclusion

**Fig. 3:**
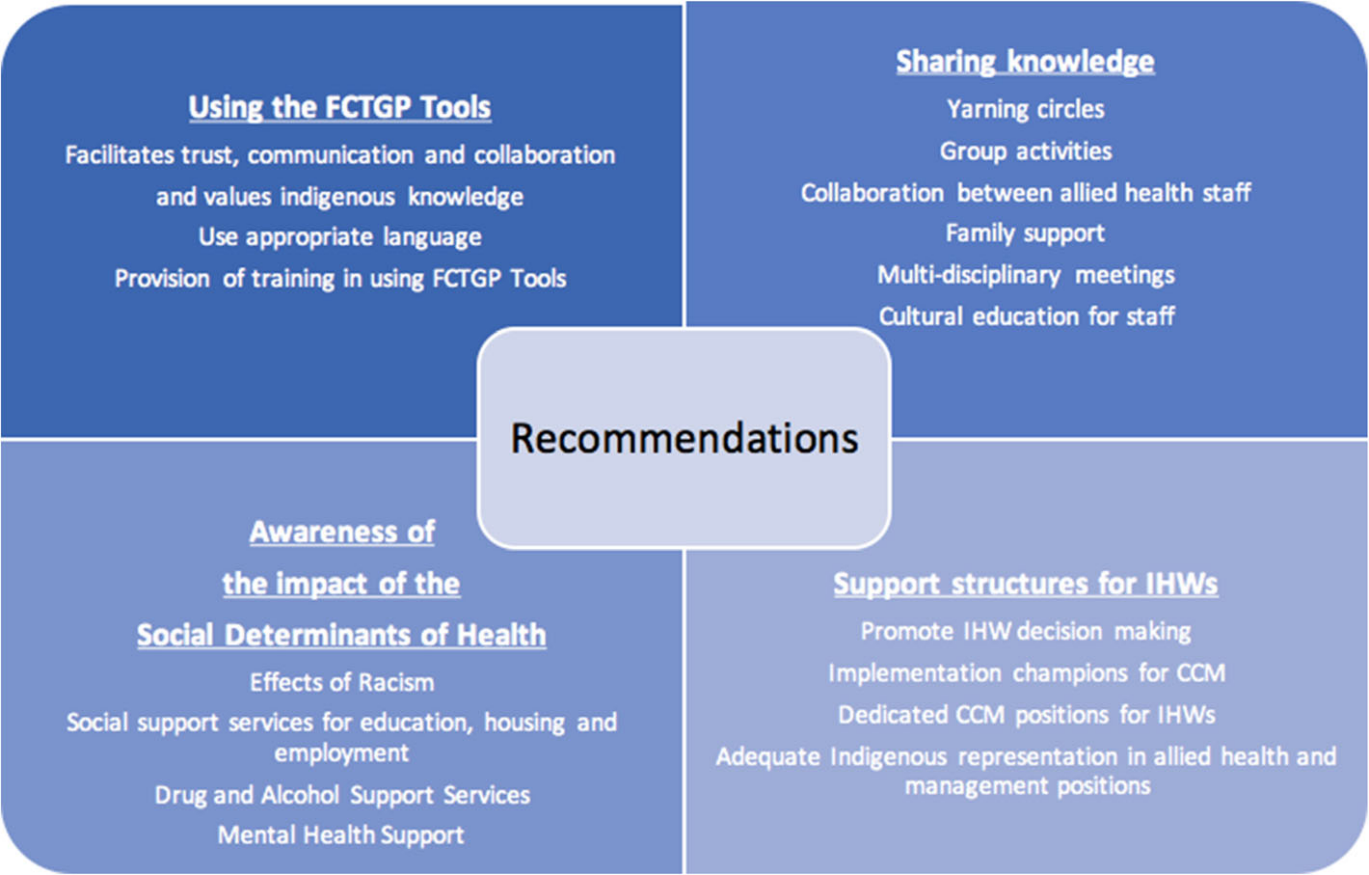
Recommendations

In regards to making recommendations for policy and practice, it is acknowledged that this study is an exploration into the perceptions of five IHWs. The findings are not intended to be generalisable; however, analytical generalisations where findings take on the form of likely implications for future endeavours in self-management are made. These are based on replications of results between cases and from the literature [33]. It should be noted that the following recommendations do not exist in isolation, as there is some interaction and interdependence between them. This means that while some recommendations may be beneficial in a particular context, it may not work in others. Because Indigenous groups are heterogeneous and exist in a dynamic context, degree to which certain ideas act as enablers or barriers could depend on the particular contexts within which they occur. A figure representing recommendations is provided (Fig. 3).

Given the reported enabling factors of the FCTGP tools; it is recommended that the Tools are utilised more broadly in Aboriginal and mainstream health services. To support this, government rebates for care plans should continue for My Health Story to provide ongoing incentive for their utilisation, with bonuses for care plan reviews to ensure continuity and follow-up. Patients with mental health conditions or drug and alcohol issues were identified as being difficult to engage in self-management tasks, however, the Flinders Tools have assisted IHWs in supporting patients with mental illness as described. A single clinical software system compatible with care plan models will aid in reducing double-data handling and improve efficacy with data entry, this would also mean that patients in transit could continue with their goals and SMS wherever they go.

It is recommended that Flinders University continue to train and support health services using the Flinders Tools for CCM, as well as training IHWs as trainers themselves within their health service, which may increase Indigenous workforce and ensure IHWs are valued and included. Ongoing social services support to Indigenous people including family, housing, and employment support could also aid in unburdening IHW roles and tackling the social determinants of the chronic conditions that afflict Indigenous peoples.

The importance of shared knowledge and family involvement in Indigenous CCM as described in this study should be harnessed. Yarning groups, walking groups and cultural activities as established core features of health services and how they interact with their communities could help aid self-management. Families may benefit from group health visits for support and as a means to provide education. These measures should lead to improved program sustainability. Cultural education to non-Indigenous Health Workers is pivotal in order to minimise discrimination and distrust, and work towards providing and maintaining culturally safe environments.

Time pressures and staff turnover negatively affects provider-client relationships by dissolving trust and weakens intervention sustainability. There should be adequate support structures for IHWs such as group meeting and debriefing sessions. Problems with the tools could be discussed with a ‘go-to’ tool champion who could be delegated by Flinders University or the clinic. Dedicated chronic condition care co-ordinator IHWs should be available to all health services. Other Allied health professionals play a positive role in patient self-care support and collaboration, however a staggered approach to referral could occur so that patients are not overwhelmed by sudden perceived bombardment of appointments.

Whilst the issues discussed in this study are not suggested to be generalizable to other contexts and international Indigenous groups, the findings of the study are arguably relevant internationally in the current climate of chronic disease burden in Indigenous people worldwide [58]. The study may provide a framework for future studies in Indigenous CCM abroad. Emerging reviews suggest that the sustainability of Indigenous CCM programs in different countries rely on similar concepts of community ownership and governance, cultural safety and supporting the role of IHWs [27].

## Abbreviations

ABS: Australian Bureau of Statistics
AIHW: Australian Institute of Health and Welfare
AMS: Aboriginal Medical Service
C&R: Cue and Response
CCM: Chronic condition management
FCTGP: Flinders Closing the Gap Program
FHBHRU: Flinders Human Behaviour and Health Research Unit
IHW: Indigenous health worker
NSW: New South Wales
P&G: Problems & goals
PIH: Partners in health
SA: South Australia
SMS: Self-management support
WA: Western Australia
WHO: World Health Organisation
